# Postoperative incidence of seizure and cerebral infarction in pediatric patients with epileptic type moyamoya disease: a meta-analysis of single rate

**DOI:** 10.1186/s41016-020-00224-y

**Published:** 2021-02-02

**Authors:** Jingjing Liu, Qinlan Xu, Hongchuan Niu, Rong Wang, Xun Ye, Xianzeng Liu

**Affiliations:** 1 Peking University International Hospital, Department of Neurology, Peking University, Beijing, China; 2 Boston Children’s Hospital, Division of Epilepsy and Clinical Neurophysiology, Department of Neurology, Harvard Medical School, Boston, USA; 3 Peking University International Hospital, Department of Neurosurgery, Peking University, Beijing, China; 4 Beijing Tiantan Hospital, Department of Neurosurgery, Capital Medical University, Beijing, China

**Keywords:** Moyamoya disease, Epileptic type, Seizure, Ischemic event, Surgery

## Abstract

**Background:**

Surgery is a conventional mature treatment for moyamoya disease (MMD). However, whether surgery is also an effective therapy for epileptic type MMD has seldom been investigated systematically. The study aims to summarize the pooled postoperative incidence of seizure and cerebral infarction in pediatric patients with epileptic type moyamoya disease.

**Method:**

The study was a systematic review and critical appraisal with a meta-analysis of cohort studies, both prospective and retrospective. Studies were identified by a computerized search of PubMed, Embase, Web of Science, Wanfang, and CNKI databases. In a literature search, a total of 7 cohort studies were identified. The *I*^2^statistic was used to quantify heterogeneity. A fixed-effect model was used to synthesize the results. The linear regression test of funnel plot asymmetry was used to estimate the potential publication bias.

**Results:**

The pooled estimated postoperative incidence of seizure in pediatric patients with epileptic type moyamoya disease was 23.44%. The pooled estimated postoperative incidence of cerebral infarction in pediatric patients with epileptic type moyamoya disease was 9.12%. Low substantial heterogeneity and potential publication bias were present.

**Conclusions:**

Evidence from this study suggests that the postoperative incidence of seizure and cerebral infarction is relatively low. Surgery is an effective and secure therapy for pediatric patients with epileptic type moyamoya disease.

## Background

Moyamoya disease (MMD) is a type of progressive occlusive cerebrovascular disease, with the significant characteristic of steno or blocked blood vessels at the end of the internal carotid artery (ICA), proximal middle cerebral artery (MCA), and anterior cerebral artery (ACA) [1]. The name “moyamoya” means “puff of smoke” in Japanese and describes the appearance of the formation of smoke-like abnormal blood vessels in the base of the skull in cerebral angiography. It frequently occurs in the East Asian population, may cause ischemic or hemorrhage stroke, epilepsy, headache, or transient ischemic attack (TIA) [2]. The epileptic seizure is the second common symptom of MMD in pediatric patients [3], and the third most common manifestation of MMD in all patients [4].MMD whose main clinical symptoms are convulsive seizures is called “epileptic type moyamoya disease” [5]. The treatment methods and clinical outcomes for this type of MMD are seldom reported.

Some of the epileptic type patients suffer seizures as the first symptom, and others may be secondary to ischemic stroke [6]. The clinical manifestation and progression of this type may be non-specific [5]. Some scholars believe that epileptic seizure in epileptic type patients is not a specific symptom but mostly caused by cerebral ischemia [7]. The cause of epilepsy in MMD could be various, including ischemic or hemorrhagic stroke [5], hyperperfusion [8], and so on. About 20–30% of MMD patients present with seizures [9], but only 3–4% epileptic type MMD without vascular events [10, 11].

Revascularization operation is effective for MMD presenting with the cerebral ischemic event has been proved by multiple studies [11–14]. Surgery could reduce the frequency of TIAs and cerebral infarction, and improve the long-term prognosis of brain functions. The cerebral hemodynamics and metabolism are also improved following surgery treatment, which could be evaluated by SPECT or PET [13]. Both direct and indirect revascularizations or the combination of these two types could obtain satisfactory results. Since epileptic type, MMD has rarely been discussed in detail before, whether surgery could improve these patients still lack research systematically. In this study, we will summarize the pooled postoperative incidence of seizure and cerebral infarction in pediatric patients with epileptic type MMD, in order to estimate the effect of the surgery treatment on these patients.

## Methods

### Literature search

**Fig. 1:**
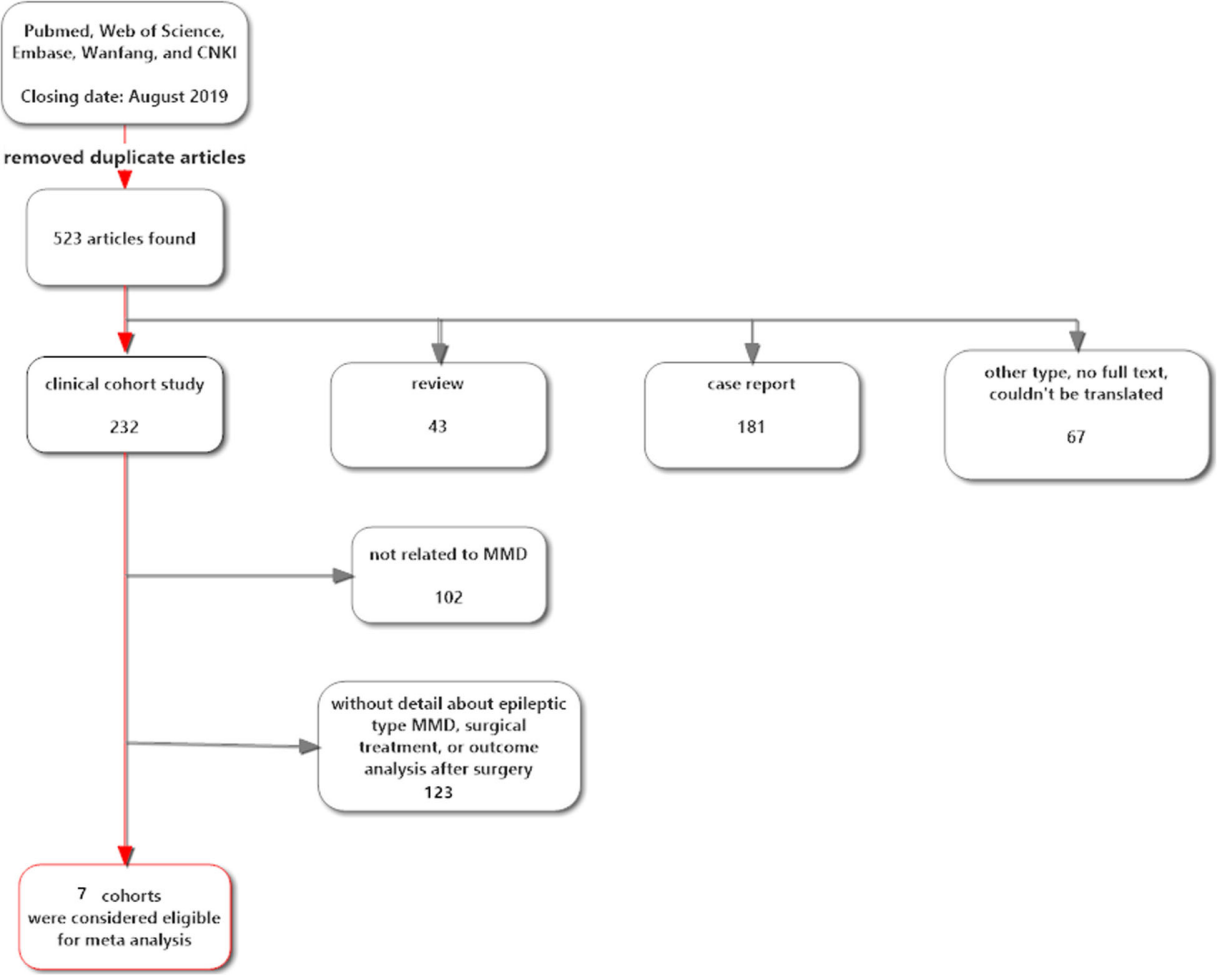
Flow chart of the literature searches for the systematic review

We computerized searched the PubMed, Web of Science, Embase, Wanfang, and CNKI databases. A combination of keywords and similar strategies was used to identify previously published studies. The medical subject heading (MeSH) key words were moyamoya disease, epilepsy or seizure, and surgery or operation. Two independent researchers (Liu J, Xu X) conducted separate literature searches. The last update for research was done in August 2019. No limitation on language was considered. After the removal of duplicate articles, a total of 523 articles were found (Fig. 1).

### Criteria for inclusion and exclusion

Articles were included in the meta-analysis if they met the following criteria: (1) prospective and retrospective cohort studies on patients with epileptic type MMD, (2) enrolled patients are younger than 18 years old, (3) at least 5 epileptic type MMD patients were included in the study, (4) chose direct and indirect revascularizations or the combination surgery treatment for these patients, (5) the prognosis of these patients after surgery was described in detail, including the postoperative incidence of seizure and cerebral infarction. We excluded all of the reviews, case reports, articles without full text, or not be translated into English or Chinese. We also excluded the studies without detail about epileptic type MMD, surgical treatment, or outcome analysis after surgery. No randomized controlled trials were found. Finally, seven cohorts [2, 6, 15–19] were considered eligible for the postoperative incidence of seizure and cerebral infarction in pediatric patients with epileptic type MMD (Fig. 1).

The following data were extracted from each study: (1) age range of the patients, (2) follow-up period, (3) other preoperative clinical manifestations except epilepsy, (4) interval between onset of clinical symptoms and surgery, (5) the modality of revascularization surgery (direct, indirect, combined), (6) angiographic and perfusion characteristics (Suzuki stage [20]), (7) postoperative complications (other than seizures and ischemic events), (8) the number of epileptic type cases, (9) the number of postoperative seizure cases, and (11) the number of postoperative cerebral infarction cases.

### Statistical analysis

Meta-analyses of a single rate were carried out by using RStudio version 3.6.1 (2019-07-05). The *I*^2^ statistic was used to quantify heterogeneity (*I*^2^ < 25%, low heterogeneity). The fixed-effects models were used to calculate an overall pooled proportion and 95% CI when the results presented as low heterogeneity. Linear regression test of funnel plot asymmetry was used to estimate the potential publication bias (*P* > 0.05, no publication bias). Influential analysis was used to detect whether there were some studies exert a very high influence on our overall results.

## Results

All of the details of the included studies are listed in Table 1.

**Table 1:** The details of the included studies

*n*	Included studies	Age range (years)	Follow-up period(years)	Preoperative clinical manifestations (except seizure)	Interval between onset of clinical symptoms and surgery	Surgery modality
1	Sainte-Rose C, et al. (2006) [15]	3.5-16	2.1-5.6	Cerebral ischemia, headache	Unknown	Indirect (multiple bur hole)
2	Choi JI, et al. (2015) [2]	2-15	2-12	Cerebral ischemia	An average of 7.4 months	Indirect (EDAS)
3	Nakase H, et al. (1993) [6]	0.4-12	0.5-17.3	No description	Within 1 year to more than 3 years	Combined (STA-MCA + EMS)
4	Ulrich PT, et al. (2011) [16]	1-18	2-25	Ischemic stroke, mental retardation, headache	Unknown	Indirect, direct, or combined (EDAS, EMS, STA-MCA + EMS)
5	Caldarelli M, et al. (2001) [17]	0.6-9	1.2-19	Hemiparesis, psychomotor retardation, malformation, fever	2-20 months	Indirect (EMS)
6	Ma Y, et al. (2018) [18]	2-18	0.5-7.5	Cerebral ischemia or hemorrhage, perfusion impairment	0.7-113 months	Indirect, direct, or combined (EDAS, bypass, burr holes)
7	Yang H, et al. (2019) [19]	3.5-16	0.2-1.2	No description	Unknown	combined
*n*	Included studies	angiographic characteristics	Postoperative complications	Epileptic type MMD cases	Postoperative seizure cases	Postoperative ischemic event cases
1	Sainte-Rose C, et al. (2006) [15]	Suzuki stage 2-4	Subcutaneous effusions (1 case)	5	2	0
2	Choi JI, et al. (2015) [2]	Suzuki stage 2-3	No description	7	1	2
3	Nakase H, et al. (1993) [6]	Suzuki stage 1-6	No description	23	4	3
4	Ulrich PT, et al. (2011) [16]	Suzuki stage 2-3	No description	15	5	Unknown
5	Caldarelli M, et al. (2001) [17]	Stenosis or occlusion of monolateral or bilateral ICA, MCA, or ACA	Epidural bleeding (1 case)	5	3	0
6	Ma Y, et al. (2018) [18]	Suzuki stage: 1-5	No description	28	7	Unknown
7	Yang H, et al. (2019) [19]	Unknown	No description	9	1	Unknown

*Abbreviations*: *EDAS* encephaloduroarteriosynangiosis, *EMS* encephalomyosynangiosis, *STA–MCA* superficial temporal artery–middle cerebral artery, *ICA* internal carotid artery, *MCA* middle cerebral artery, *ACA* anterior cerebral artery, *TIA* transient ischemic attack

Suzuki stage [20]: stage 1 no abnormal findings can be observed except for carotid fork stenosis; stage 2 the stenosis has progressed at the carotid fork; the moyamoya vessels can be observed and all the main cerebral arteries are dilated; stage 3 the moyamoya vessels develop on the base of the brain and its constituent vessels are comparatively thick and rough with the anterior or middle cerebral arteries missing; stage 4 the moyamoya vessels become thin with the anterior and middle cerebral arteries missing; stage 5 the moyamoya vessels contract to the base of the brain and even the posterior cerebral arteries disappear; stage 6 the intracranial internal carotid artery is not visualized; the intracerebral vessels are perfused with blood from the external carotid artery and/or vertebral artery.

**Fig. 2:**
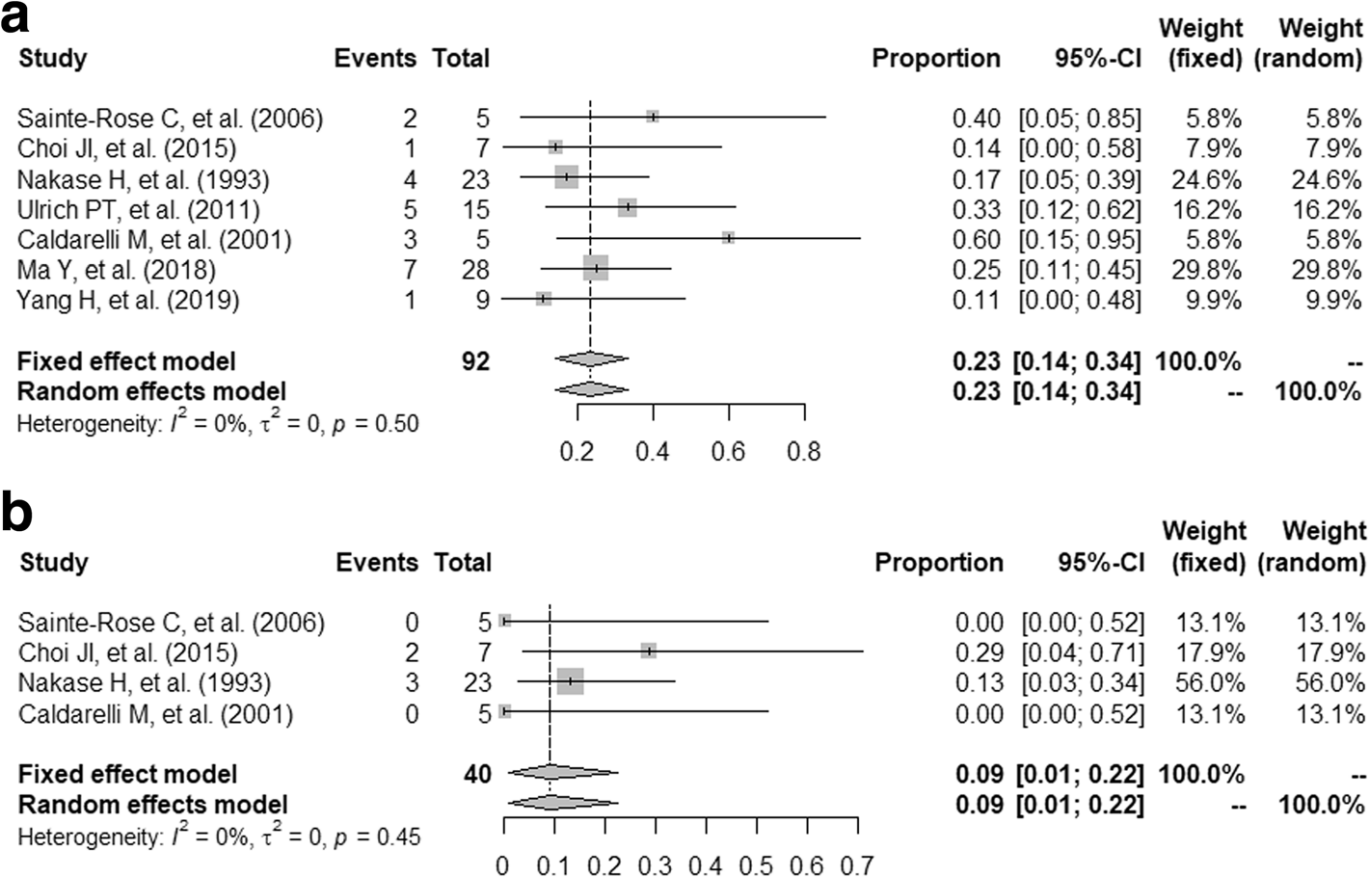
forest plot of postoperative incidence. **a** Forest plot of postoperative incidence of seizure in patients with epileptic type MMD. **b** Forest plot of postoperative incidence of cerebral infarction in patients with epileptic type MMD

**Fig. 3:**
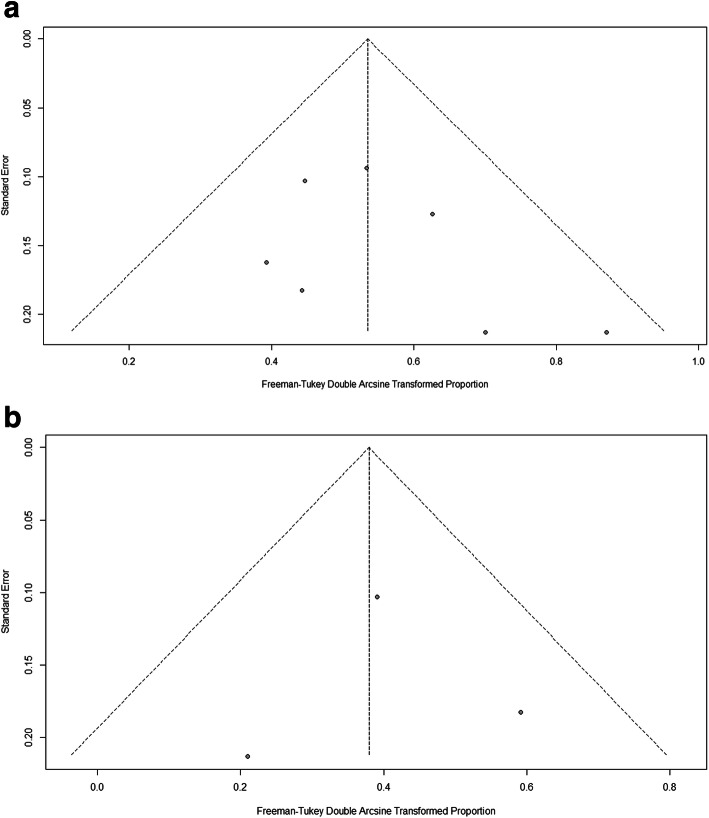
Funnel plot of postoperative incidence. **a** Funnel plot of postoperative incidence of seizure in patients with epileptic type MMD. **b** Funnel plot of postoperative incidence of cerebral infarction in patients with epileptic type MMD

The summarized pooled postoperative incidence rate of seizure in pediatric patients with epileptic type MMD in the seven studies using the fixed-effects models was 23.44% (95% CI 14.39–33.63%) with low heterogeneity (Tau^2^ = 0, *I*^2^ = 0% (95%CI 0–67.2%), *P* = 0.5) (Fig. 2 a). Low publication bias was estimated by the linear regression test of funnel plot asymmetry (*P* = 0.33) (Fig. 3a).

The summarized pooled postoperative incidence rate of seizure in pediatric patients with epileptic type MMD would change ≤ 2.6% after the exclusion of any studies. The maximum increase of the rate would be 2.6% after the exclusion of the study by Nakase H, et al. (1993) [6], whereas the maximum reduction would be 1.9% after the exclusion of the study by Ulrich PT, et al. (2011) [16]. Removing any of the findings of these studies will not make the heterogeneity of the studies significant (*I*^2^ = 0%).

The summarized pooled postoperative incidence rate of cerebral infarction in pediatric patients with epileptic type MMD in the four studies using the fixed-effects models was 9.12% (95% CI 0.75–22.34%) with low heterogeneity (Tau^2^ = 0, *I*^2^ = 0% (95% CI 0–82.5%), *P* = 0.45) (Fig. 2 b). Low publication bias was estimated by the linear regression test of funnel plot asymmetry (*P* = 0.70) (Fig. 3b).

The summarized pooled postoperative incidence rate of cerebral infarction in pediatric patients with epileptic type MMD would change ≤ 3.2% after the exclusion of any studies. The maximum increase of the rate would be 2.4% after the exclusion of the study by Sainte-Rose C, et al. (2006) [15] or Caldarelli M, et al. (2001) [17], whereas the maximum reduction would be 3.2% after the exclusion of the study by Choi JI, et al. (2015) [2]. Removing any of the findings of these studies will not make the heterogeneity of the studies significant (*I*^2^ = 0%).

## Discussion

The management of MMD includes medical treatment and surgical treatment. Platelet aggregation inhibitors [21]or calcium channel blockers [21, 22] may generate excellent but transient effects, could be only applied to mild cases [17] or acute phase of stroke [11]. Surgical treatment is considered to be the most effective method to treat MMD, especially for MMD, manifesting as a cerebral ischemic symptom [11]. However, few studies focus on the surgical treatment of epileptic type MMD, and there is a lack of randomized controlled studies and meta-analysis. Thus, we reviewed the current literature and summarized the efficacy of surgical treatment for epileptic type MMD.

We calculated an overall pooled postoperative incidence of seizure in pediatric patients with epileptic type MMD, which was 23.44%. Most of the patients with epileptic type MMD suffered seizures after surgery could be controlled by anti-epileptic drugs [15–17], and only very few patients deteriorated or without improvement after surgery [2, 18]. We also calculated the overall pooled postoperative incidence of cerebral infarction in pediatric patients with epileptic type MMD, which was 9.12%. The data suggest that surgical revascularization is a secure intervention for pediatric epileptic type MMD and most treated patients could gain symptom improvement [23].

The risk factors for epilepsy and ischemic event after cerebral revascularization in pediatric patients with epileptic type MMD were various. Age of patients, course of clinical symptoms, and severity of clinical presentations may all affect the prognosis of operation [6, 18, 19]. Only one study [18] explored the risk factor of seizure recurrence in epileptic type MMD. They identified the duration of epilepsy as an independent risk factor for recurrent seizure after surgery in pediatric patients with epileptic type MMD. Nevertheless, the intervals between onset of clinical symptoms and surgery in our study were not consistent; it is particularly important to group analysis by different course in the future. In addition, age less than 1 year and severe abnormal imageological findings correlate with poor prognosis of epileptic type MMD [3], infantile onset and severe clinical manifestations are more likely to have recurrent seizures or cerebral infarction after surgery. In our study, although the age range and angiographic characteristics were not consistent among the included studies, the pooled rates still showed low heterogeneity. Hence, group analysis should be considered according to different ages and severity of clinical presentations in the future.

Most scholars believed that seizure could be associated with ischemia [19]. Among the studies included in our meta-analysis, it is hard to identify whether the preoperative seizures came from ischemia events, due to epilepsy and ischemic event always exist together and has an analogical progression in most of the patients with MMD [6]. Choi et al. [2] reported that surgery could prevent epileptic seizures and obtained more approving clinical outcomes when applied to patients with epileptic type MMD compared to ischemic type MMD, but there were no differences in postoperative neuroimaging and hemodynamic changes between the two groups. Yang et al. [19] did not consider the presence of epileptic symptoms before surgery is a risk factor for postoperative epileptic recurrence. The different outcomes between epileptic type and ischemic type need more future studies.

Different operation procedures (indirect, direct, or combined) may affect the prognosis of MMD. Indirect revascularization is widely used in pediatric patients because of its simple procedure and few complications [24–26]. Many studies confirmed the effectiveness of this modality, but direct revascularization can improve cerebral blood flow perfusion immediately as its advantage [25, 26]. Therefore, more and more surgeons chose combined revascularization to treat patients with MMD [27, 28], just as most of the included studies [6, 16, 18, 19] in our meta-analysis. However, in our analysis, different modalities did not show obvious different postoperative incidences of seizure and cerebral infarction, which was in line with the finding of Ma Y et al. [18], who believed that the surgical modalities would not affect the outcome of the surgery. Further research on the effect of the different operation techniques is needed.

There are many possible causes of postoperative seizure in MMD patients: ischemic events, intracranial hemorrhage, hyperperfusion syndrome, poor scalp healing and infection, surgical procedure [29]. Seldom studies could identify where the postoperative seizure came from. No postoperative hyperperfusion mentioned in the included studies. Only two included studies described the postoperative complications of patients with epileptic type MMD. In the study by Sainte-Rose C, et al. [15], one patient suffered subcutaneous cerebrospinal fluid effusions and had a single seizure 5 months postoperatively. Due to such a long interval and no infection occurred, this seizure was unlikely caused by subcut effusion. In the study by Caldarelli M, et al. [17], one patient experienced epidural bleeding during the surgery, and died 6 months later. This patient presented seizure postoperation, but controlled by medication well. It is hard to say whether the seizure was induced by this postoperative complication. But omitting this study will not change the pooling results in our analysis.

There were several limitations in our study: the numbers of patients and studies were all tiny, and all of these studies were retrospective and non-randomized. That may because the incidence of epileptic type MMD is relatively low, and few scholars focus on this type of MMD. Follow-up periods were not consistent among individuals, ranging from 0.2 to 25 years, which implies that the observational time of some patients may not be sufficient. Most of the studies did not describe the detail of the clinical feature of this type of patients, which results in the difficulty of distinguishing whether ischemic events or other reasons cause the patient’s preoperative and postoperative seizures. In a word, future studies should focus on the epileptic type MMD in large-scale randomized controlled clinical studies.

## Conclusions

Evidence from this study suggests that the postoperative incidence of seizure and cerebral infarction is relatively low. Surgery is an effective and secure therapy for pediatric patients with epileptic type moyamoya disease.

## Data Availability

All data generated or analyzed during this study are included in these published articles.

## Abbreviations

MMD: Moyamoya disease
ICA: Internal carotid artery
MCA: Middle cerebral artery
ACA: Anterior cerebral artery
TIA: Transient ischemic attack
EDAS: Encephaloduroarteriosynangiosis
EMS: Encephalomyosynangiosis
STA–MCA: Superficial temporal artery–middle cerebral artery
